# Quantitative Detection of Thitsiol and Urushiol as Markers from the *Gluta usitata* Lacquer Tree Using HPLC

**DOI:** 10.3390/molecules29010149

**Published:** 2023-12-26

**Authors:** Youngseo Lee, Jihye Lee, Kang-Bong Lee, Won-Yong Lee, Yeonhee Lee

**Affiliations:** 1 Advanced Analysis and Data Center, Korea Institute of Science and Technology, Seoul 02792, Republic of Korea; gksten@kist.re.kr (Y.L.); 023461@kist.re.kr (J.L.); 2 Department of Chemistry, Yonsei University, Seoul 03722, Republic of Korea; wylee@yonsei.ac.kr; 3 Green City Technology Institute, Korea Institute of Science and Technology, Seoul 02792, Republic of Korea; leekb@kist.re.kr

**Keywords:** traditional lacquer, thitsiol, urushiol, CNSL, HPLC, quantitative analysis

## Abstract

Lacquer sap has been traditionally used in coatings and artwork. Suitable types of lacquer are required to preserve and restore artifacts. Recently, unsuitable cashew nut shell liquid (CNSL) has often been mixed with lacquer sap, so it is necessary to identify the characteristics of lacquer sap by the production area. However, research is still focused on urushiol and laccol. In this study, Myanmarese lacquer sap collected from *Gluta usitata*, which contains thitsiol as the main component, was analyzed by HPLC to quantify thitsiol using the standards 3-(10-phenyldecyl) benzene-1,2-diol (thitsiol 16) and 3-(8Z,11Z-pentadecadienyl)-benzenediol (urushiol 15:2) as markers, and calibration curves were plotted. The coefficients of determination (R^2^) for thitsiol 16 and urushiol 15:2 were 0.9985 and 0.9983, respectively. In addition, a blind test was conducted to confirm that accurate quantitative analysis was possible even when Myanmarese lacquer was mixed with lacquer from another production area, which contained urushiol as the main component, and CNSL, which contained cardol, a completely different catechol. Quantitative analysis of thitsiol 16 and urushiol 15:2 in Myanmarese lacquer using HPLC can be used to evaluate the quality of lacquer sap and for more sophisticated activities such as restoration by classifying differences in lacquer sap by the production area.

## 1. Introduction

Traditional lacquer is very durable and glossy, which has made it a popular coating and adhesive for ceramics, wood, and metals for thousands of years [1]. Traditional lacquer has been widely used in East Asia, including Korea, China, Japan, Vietnam, and Myanmar. Lacquer is deeply intertwined with the cultural heritage of these countries [2,3,4,5,6]. It is also widely used in modern times, from household items such as plates, bowls, and spoons to artwork. It is desirable to be able to differentiate between different types of lacquer for the preservation and restoration of cultural artifacts.

Traditional lacquer is obtained by collecting lacquer sap from specific trees and filtering the sap to remove impurities [7]. Traditional lacquer is primarily produced in Asia. Some examples of lacquer trees include *Toxicodendron vernicifluum* (*T. vernicifluum*) in Korea, Japan, and China; *Toxicodendron succedaneum* (*T. succedaneum*) in Taiwan and Vietnam; and *Gluta usitata* (*G. usitata*) in Myanmar [8,9,10,11,12,13]. Lacquer sap from these trees contains catechols, water, lipids, proteins, and other lacquer components. Specifically, urushiol is the catechol from *T. vernicifluum*, laccol is from *T. succedaneum*, and thitsiol is from *G. usitata*. Urushiol and laccol primarily consist of 3-pentadecyl catechol and 3-heptadecyl catechol, respectively, while thitsiol contains phenyldodecyl catechol and heptadecyl catechol as the main components. A coating material can also be derived from cashew trees, specifically from the shells of cashew seeds. This substance is known as cashew nut shell liquid (CNSL), with cardol being the main component. CNSL is often used as a substitute for traditional lacquer because of its lower cost. However, CNSL contains anacardic acid, which can cause skin irritation, allergic reactions, and respiratory issues when inhaled [14,15]. Improper disposal of CNSL can lead to environmental pollution. Additionally, CNSL has different drying properties due to its chemical composition compared to traditional lacquer. Mixing a traditional lacquer sap with CNSL may not completely reproduce the gloss and clarity and may result in incomplete hardening of lacquer coatings. This mixing is particularly problematic in Asia, where lacquer-based cultural artifacts are prevalent, as it may hinder the preservation of materials during restoration processes. Thus, the use of CNSL in processes that require traditional lacquer should be carefully evaluated.

Lacquer dries at temperatures ranging from 20 °C to 25 °C and at a relative humidity of 70–80%. Moisture evaporates during the drying process and major components, including catechols, undergo oxidation and polymerization to form crosslinked bonds, causing the material to harden. The hardened lacquer film is glossy, highly durable, chemical- and moisture-resistant, and insect repellent because of its complex molecular composition and limited solubility [16,17,18,19]. Previous studies have used various techniques to analyze lacquer sap and lacquered cultural heritage artifacts, such as pyrolysis-gas chromatography-mass spectrometry (Py-GC/MS) [18,19,20], surface analysis using time-of-flight secondary ion mass spectrometry (ToF-SIMS) [21,22], and X-ray photoelectron spectroscopy (XPS) [23,24] to understand the structural properties and composition of lacquer. However, few quantitative studies of mixed lacquer have been performed [20,25]. In our previous study, the chemical structures of Korean, Chinese, and Vietnamese lacquer films and CNSL films were characterized using ToF-SIMS for the first time. The main components of lacquer by production area were identified and the complexes of major components were also identified. In addition, the copolymerization of characteristic catechols, namely urushiol and laccol, by radical transfer of blended lacquer sap was observed using ToF-SIMS. Thus, the possibility of quantitative analysis using ToF-SIMS was suggested. The characteristic components and pyrolytic products of specific catechols of lacquer were observed using Py-GC/MS. Py-GC/MS analysis of mixed lacquer resulted in high quantitative detection with no matrix effect. HPLC was used to determine the contents of urushiol and laccol in Korean, Chinese, Japanese, and Myanmarese lacquer and CNSL to provide quantitative information on lacquer sap. And the reliability of the analysis method was verified by conducting a blind test with urushiol 15:1, 15:2, and 15:3 as markers. Since the previous study did not quantify thitsiol, the main component of Myanmarese lacquer sap, this study aims to provide exact quantitative information for the restoration of lacquered cultural remains using specific markers for the component thitsiol. This information will help improve the knowledge of the physical and chemical properties of lacquer and allow identification of lacquer according to its production area.

HPLC analysis is a commercially available and accessible method for the quantitative analysis of lacquer molecules [26,27]. HPLC allows the precise and accurate quantification of lacquer components. By establishing calibration curves using HPLC analysis results, the exact concentration of specific compounds can be determined. Quantitative data are useful for quality control purposes and gaining insight into lacquer composition and characteristics. HPLC is a highly sensitive and selective technique, enabling the detection and separation of various compounds even at low concentrations, making it possible to identify and quantify specific markers. Furthermore, when analyzing lacquer samples with different key components, HPLC can facilitate the study and comparison of lacquer from objects with diverse cultural contexts.

In the present study, the main component of Myanmarese lacquer, thitsiol, was used as a marker. Thitsiol and urushiol in Myanmarese lacquer were quantified, and a calibration curve for thitsiol was constructed using HPLC. HPLC analysis has the disadvantage of requiring pretreatment compared to other surface analysis equipment, but it is advantageous in analyzing the basic components of lacquer because it is highly accessible compared to surface analysis instruments. In order to apply HPLC to the analysis of lacquer sap, centrifugation and filtering were used for pretreatment of the sample to exclude lipid components other than catechol as much as possible. This method is useful for identifying catechol when analyzing lacquer by HPLC. In addition, by individually mixing Korean lacquer, Japanese lacquer, and CNSL with Myanmarese lacquer and constructing calibration curves, the composition of mixed lacquer with unknown components was estimated using the concentration of thitsiol in *G. usitata*. A blind test was conducted to indicate the reliability of the research findings. Quantitative analysis using HPLC can contribute to the use of desired materials and techniques in modern lacquer products and help to maintain quality, especially for use in traditional lacquer crafts. Quantitative studies of mixed lacquer sap are necessary for geographical origin identification, tracking cultural and historical materials, and assurances of production quality.

## 2. Results and Discussion

### 2.1. Quantitative Analysis of Lacquer Using HPLC

Lacquer samples contain different main catechol components depending on the type of tree used as the raw material. The primary component of Myanmarese lacquer is thitsiol, which is obtained from *G. usitata*. The lacquer sap used in Korea and Japan is obtained from *T. Vernicifluum* and its primary component is urushiol. CNSL is derived from *Anacardium occidentale* and is used as a substitute for lacquer because of its similar characteristics. However, CNSL contains formalin, which is harmful to the human body. Lacquer is a complex mixture containing various catechols, proteins, and water, which makes quantitative analysis challenging. Therefore, the present study focused on a single molecule, the main catechol component present in lacquer sap, which was used as a marker. Thitsiol is the main component of Myanmarese lacquer sap, while urushiol is the main component of Korean lacquer sap. The selected markers for this study are described in the Materials and Methods section.

**Figure 1:**
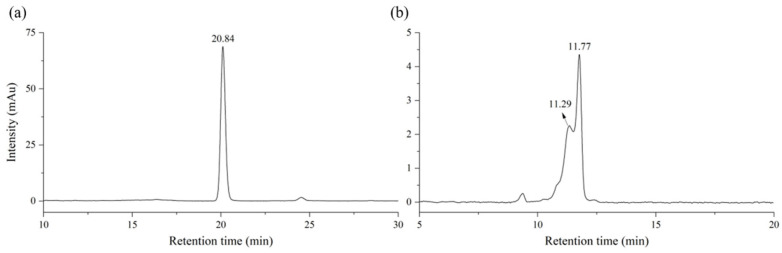
HPLC chromatograms of standards: (**a**) thitsiol 16 and (**b**) urushiol 15:2.

To determine the main components of the lacquer sap, standard samples were initially analyzed and used as markers. Commercially available standard samples were used. Figure 1 presents HPLC chromatograms of the standards according to each analysis condition. Thitsiol [3-(10-phenyldecyl) benzene-1,2-diol, thitsiol 16] exhibited a peak corresponding to 10-phenyldecyl catechol with a retention time of 20.84 min, using a 280 nm UV detector. Different analysis methods were used for each standard solution and they are described in Section 3. Urushiol showed double peaks corresponding to 3-pentadecyl catechol with retention times of 11.29 and 11.77 min, using a 260 nm UV detector. As shown in the previous study, split peaks were observed [25]. The splitting of the peak might originate from the chemical structure of urushiol 15:2, including two double bonds and two phenol groups. The retention time, response factor, limit of detection (LOD), and limit of quantification (LOQ) are summarized in Table 1. LOD and LOQ were determined with 5 ppm standard solutions, which produced signal-to-noise ratios larger than 3.3 and 10, respectively. The response factor, LOD, and LOQ of thitsiol 16 were 20.14, 0.24 ppm, and 0.79 ppm. And the response factor, LOD, and LOQ of urushiol 15:2 were 17.48, 0.38 ppm, and 1.26 ppm, respectively.

**Figure 2:**
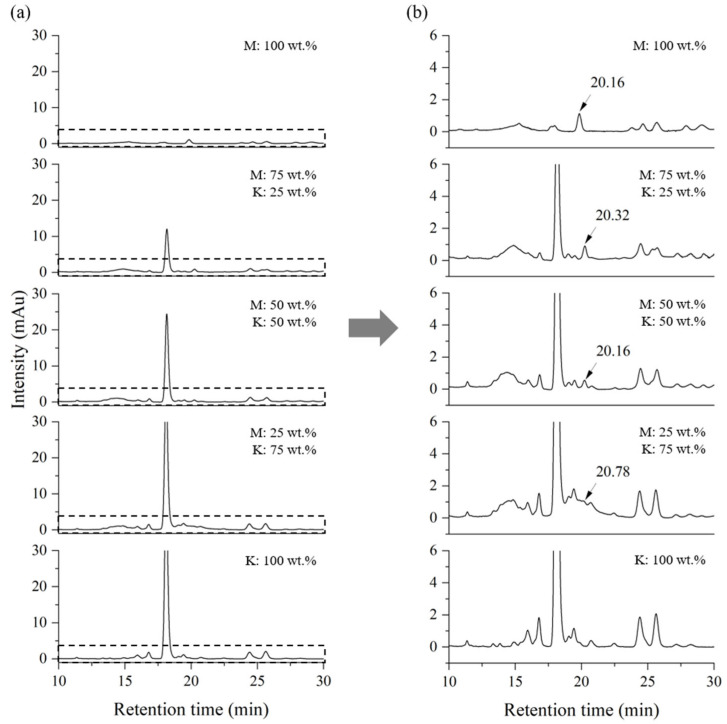
HPLC chromatograms of blended Myanmarese (M) and Korean (K) lacquer samples analyzed under thitsiol analysis conditions. (**a**) Full HPLC chromatograms and (**b**) magnified chromatograms.

For the quantification of thitsiol 16 and urushiol 15:2 in Myanmarese lacquer, chromatograms were obtained under thitsiol analysis conditions after mixing by weight ratio Korean lacquer samples, which contained urushiol as the main component, and Myanmarese lacquer samples, which had thitsiol as the main component, and their chromatograms are shown in Figure 2. The magnified peak in Figure 2b showed relatively higher intensity compared with the other peaks and was consistent with the standard sample. This sample contained thitsiol 16, suggesting that this peak could be used as a marker for Myanmarese lacquer. As the ratio of Myanmarese lacquer decreased, the intensity of this peak also decreased.

Table 2 shows the quantitative values of thitsiol 16 and urushiol 15:2 components in Myanmarese and Korean blended samples using the response factor shown in Table 1. The standard deviation was obtained from five repeated analyses. The contents of thitsiol 16 in Myanmarese and Korean samples were 7% and approximately 0.2%, respectively, which was consistent with previously reported results [28,29].

**Figure 3:**
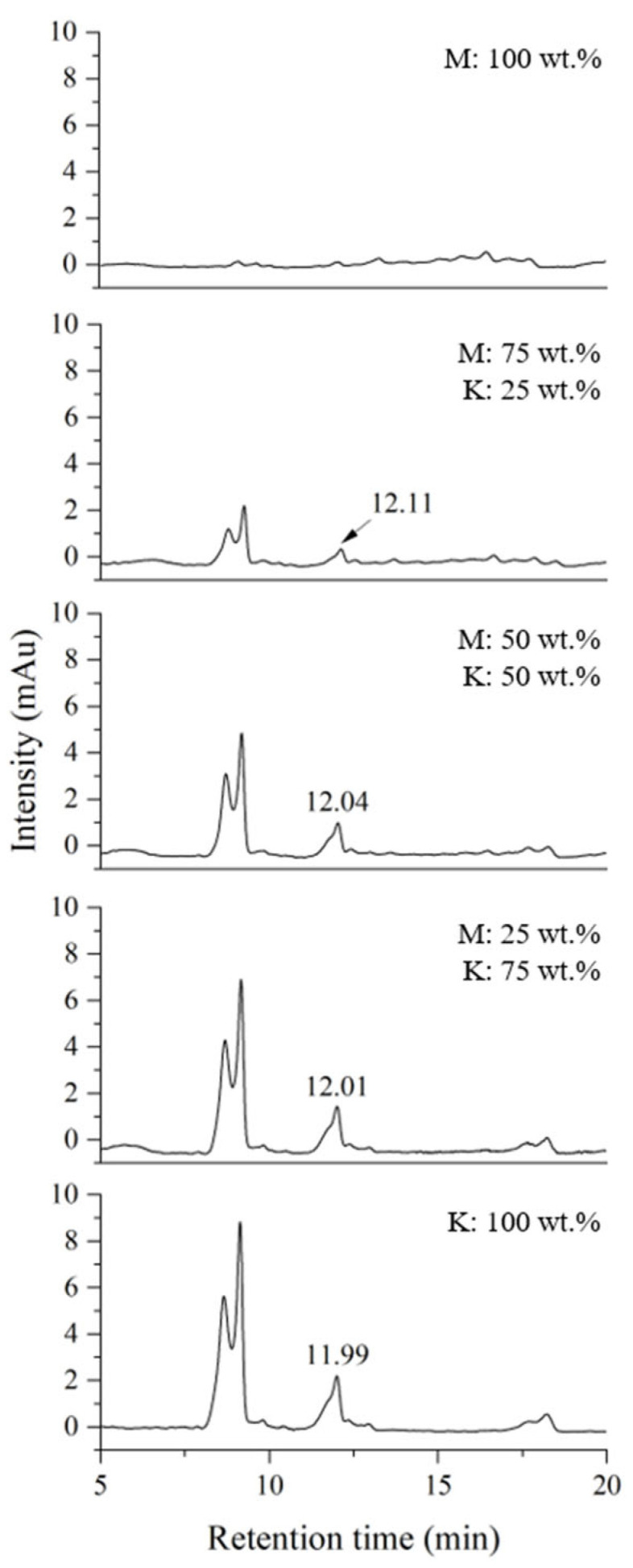
HPLC chromatograms of blended Myanmarese (M) and Korean (K) lacquer samples analyzed under urushiol analysis conditions.

Figure 3 shows the chromatograms obtained under urushiol analysis conditions by mixing Myanmarese and Korean lacquer samples at the same weight ratio as shown in Figure 2. The double peak between 8 and 9 min was the peak for urushiol 15:3 and the peak at approximately 12 min corresponded to urushiol 15:2. The peaks for urushiol 15:2 and 15:3 were separate, indicating that the HPLC analysis conditions were suitable for the desired analysis. The higher the proportion of Korean lacquer sap, the larger the area of the peak at a retention time of approximately 12 min. The content of urushiol was very low in the Myanmarese lacquer. Using the HPLC experimental conditions for urushiol 15:2 resulted in an unresolved peak for urushiol in blended lacquer samples.

**Figure 4:**
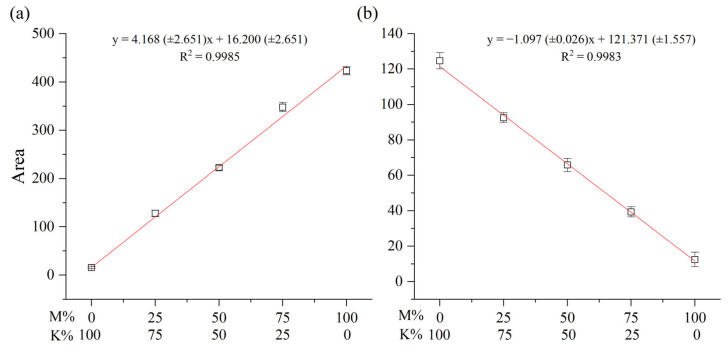
Calibration curves of blended Myanmarese (M) and Korean (K) lacquer samples analyzed under (**a**) thitsiol analysis conditions and (**b**) urushiol analysis conditions.

After repeatedly analyzing the mixture of Myanmarese and Korean samples five times, calibration curves using the areas of the thitsiol 16 and urushiol 15:2 peaks were plotted, as shown in Figure 4. The slope of the thitsiol 16 calibration curve was 4.168, the intercept was 16.200, and the coefficient of determination (R^2^) was 0.9985, indicating relatively high linearity. Similarly, Figure 4b shows the calibration curve of urushiol 15:2 analyzed under urushiol analysis conditions and with urushiol 15:2 serving as a marker, the peak area increased as the weight ratio of the Korean lacquer sample increased. The calibration curves of the lacquer mixtures demonstrated high linearity. Both figures show the concentration of Myanmarese samples on the *x*-axis. These obtained calibration curves could be used to estimate the amount in another unknown lacquer sap.

### 2.2. Blind Test for Quantification of a Mixed Lacquer Sap

**Figure 5:**
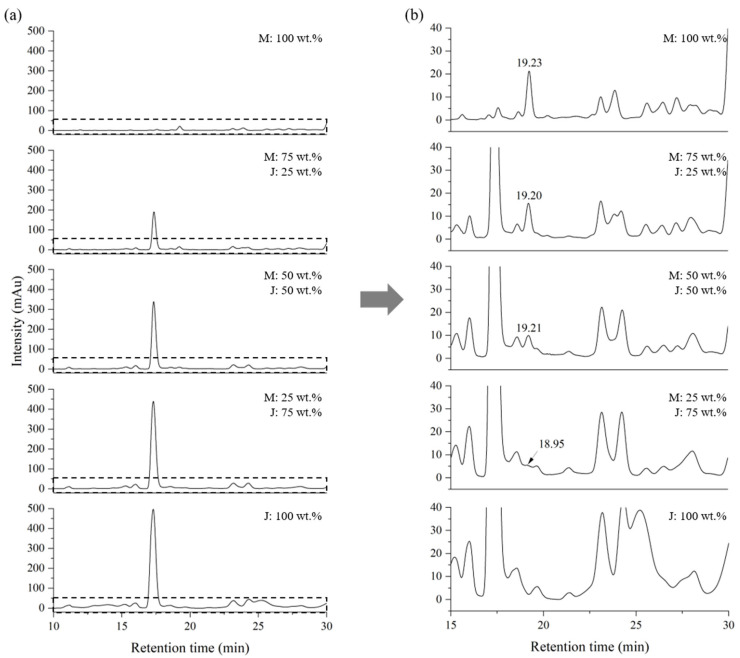
HPLC chromatograms of blended Myanmarese (M) and Japanese (J) lacquer samples analyzed under thitsiol analysis conditions. (**a**) Full HPLC chromatograms and (**b**) magnified chromatograms.

**Figure 6:**
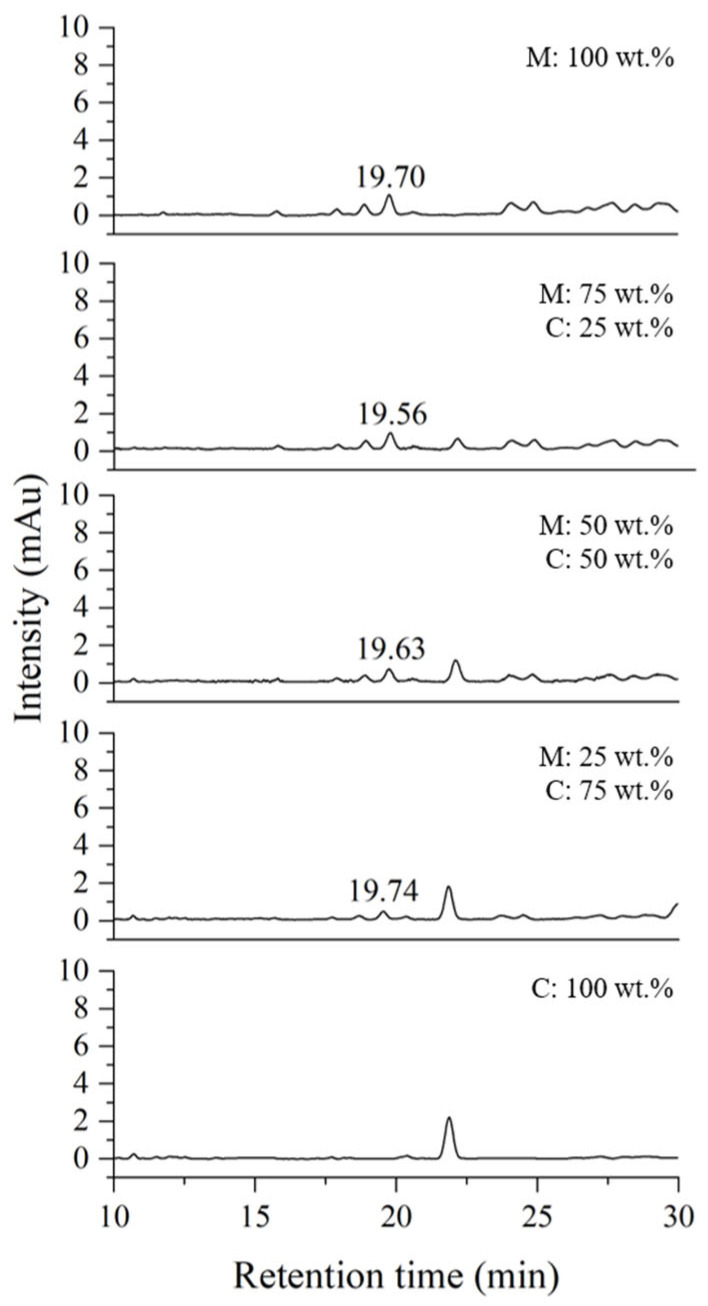
HPLC chromatograms of blended Myanmarese (M) lacquer and CNSL (C) samples analyzed under thitsiol analysis conditions.

To verify the quantitative experiment, a blind test was conducted under the conditions of thitsiol analysis using Myanmar–Japan lacquer (MJ) and Myanmar–CNSL (MC) blended samples. The mixing ratios used to plot the calibration curve are shown in Table 3. The MJ blended samples, which contained urushiol as the main component, similar to the Korean lacquer samples, were used to determine whether the Japanese lacquer samples had a different effect in the HPLC analysis. The MC blended samples were analyzed to determine the reliability of the results by examining samples with a completely different chemical composition. To establish calibration curves for quantitative analysis, samples of the MJ and MC blended samples were analyzed using HPLC under thitsiol analysis conditions and their chromatograms were obtained as shown in Figure 5 and Figure 6, respectively. The thitsiol 16 peak was determined from the retention time. Both Figure 5 and Figure 6 show that there was a decrease in the area of the thitsiol 16 peak at approximately 19 min. In Figure 5, there is a prominent peak with a retention time of approximately 17 min that gradually increased in area as the amount of Japanese lacquer sap increased. This peak was thought to be the urushiol peak, which exhibited a small shift because the major urushiol in Japanese lacquer sap had a slightly different composition from that in Korean lacquer sap. Figure 6 shows the chromatograms of the MC blended samples. It can be seen that the peak at about 22 min increased with each increase in the ratio of CNSL. This was estimated to be the peak of anacardic acid, one of the main components of CNSL [30].

**Figure 7:**
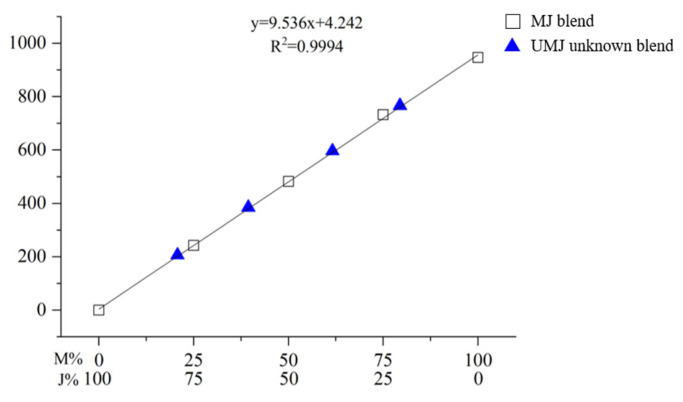
The calibration curve of blended Myanmarese and Japanese lacquer samples analyzed under thitsiol analysis conditions. The blue triangles indicate UMJ (unknown blend of Myanmar–Japan lacquer).

**Figure 8:**
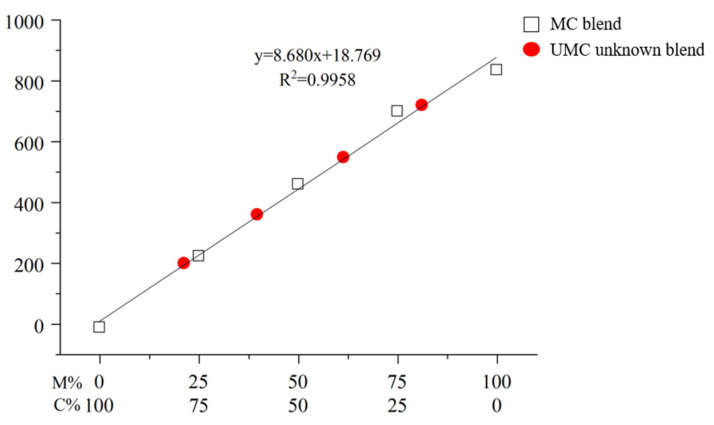
The calibration curve of blended Myanmarese lacquer and CNSL analyzed under thitsiol analysis conditions. The red circles indicate UMC (unknown blend of Myanmar–CNSL lacquer).

The calibration curves were drawn with the area of the main peak representing thitsiol 16 in Figure 5 and Figure 6 on the *y*-axis and the concentration of the blended lacquer samples on the *x*-axis. Figure 7 shows the calibration curve of the blended Myanmarese and Japanese lacquer samples. The R^2^ value was 0.9994, showing high linearity. The regression equation was y = 9.536x + 4.242. Figure 8 shows the calibration curve of the blended Myanmarese lacquer and CNSL samples analyzed under thitsiol analysis conditions. The regression equation was y = 8.680x + 18.769 and the R^2^ value was 0.9958. Both calibration curves had high linearity and high accuracy for quantitative results because the curves had R^2^ values close to 1. Thus, using HPLC, it was possible to quantitatively analyze various lacquer samples according to the area of lacquer production.

**Figure 9:**
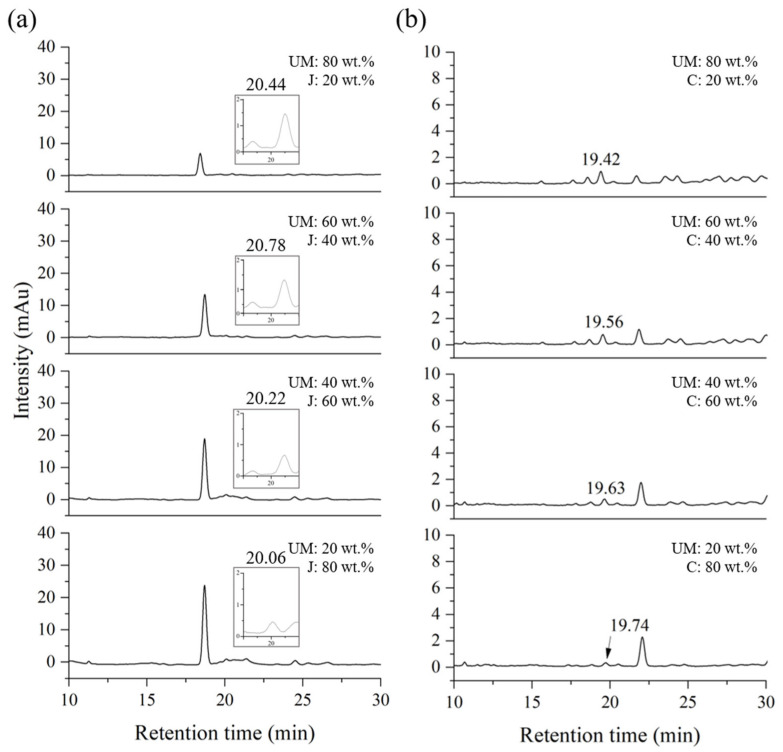
HPLC chromatograms of unknown blended samples. (**a**) UMJ01–UMJ04 and (**b**) UMC01–UMC04. UM, unknown Myanmarese lacquer; J, Japanese lacquer; C, CNSL.

One researcher prepared Myanmar–Japan blended samples (UMJ unknown blend samples) and Myanmar–CNSL blended samples (UMC unknown blend samples), while the other researchers analyzed them without knowing the exact mixing ratio. Figure 9 shows the chromatograms of UMJ and UMC unknown blended samples. In the case of UMJ unknown blended samples, the peak of thitsiol 16 was detected at about 20.5 min. For UMC unknown blended samples, the peak of thitsiol 16 was detected at about 1.95 min. An UMJ unknown blended sample and UMC unknown blended sample were prepared and HPLC analysis was performed under thitsiol analysis conditions to obtain the thitsiol 16 peak. By substituting the area of this peak into each calibration equation, the concentration of thitsiol 16 in Myanmar lacquer was calculated, as shown in Figure 7 and Figure 8. The unknown samples were plotted on the calibration curve, with a blue triangle for the UMJ unknown blended sample and a red circle for the UMC unknown blended sample, respectively. For the UMJ unknown blended sample, the highest error compared with the content of the actual blended lacquer solution was ±1.35% and the lowest value was ±0.17% (Table 4). In the case of the UMC unknown blended sample, the highest error was ±0.38% and the lowest error was ±0.21%. Accordingly, the content of Myanmar lacquer in the unknown sample was accurately quantified and precise quantification of the unknown lacquer sample was obtained using a standard. In addition, there was no interference between the lacquer sample containing urushiol and the lacquer sample containing thitsiol, and accurate quantification was obtained even though other catechol compounds were present. The results of the blind test of unknown Myanmarese lacquer samples verified the reproducibility of the thitsiol analysis conditions, and the optimal experimental conditions were established even when applied to actual lacquer samples.

## 3. Materials and Methods

### 3.1. Materials

Myanmarese, Korean, and Japanese lacquer sap were collected from *G. usitata* (Myanmarese lacquer sample) and *T. vernicfluum* (Korean and Japanese lacquer samples) trees and filtered using traditional paper. Korean lacquer was collected from Wonju by Lee Hyung-man, a human cultural asset. Japanese and Myanmarese lacquer were purchased from Pyeonghwa Shell (Seoul, Korea). CNSL was purchased from Dongbang Cashew (Jincheon, Korea). The standards for thitsiol 16 [3-(10-phenyldecyl) benzene-1,2-diol]] and urushiol 15:2 [3-(8Z,11Z-pentadecatrienyl)-1,2-benzenediol] were purchased from Phytolab GmbH (Vestenbergsgreuth, Germany). The structural formulae of the standard compounds are shown in Table 5.

### 3.2. Methods

#### HPLC

HPLC (1290 Infinity instrument, Agilent Technologies, Santa Clara, CA, USA) was used to quantify the contents of thitsiol and urushiol 15:2 in lacquer samples. The column used was a C18 reversed-phase column (YMC-Pack Pro, 4.6 mm I.D., S-5 µm, 12 nm, YMC CO., Kyoto, Japan). Different analysis conditions were employed for the quantification of thitsiol 16 and urushiol 15:2, as summarized in Table 6. In summary, a 0.5 wt.% solution was prepared by dissolving a lacquer sample in solvent (acetone for thitsiol analysis and chloroform for urushiol analysis), followed by vortexing for 5 min. Centrifugation was performed at 10,000 rpm for 1 h and the solution was filtered through a 0.45 µm polytetrafluoroethylene filter to remove settled foreign substances. Subsequently, the solution was diluted with solvent to a concentration of 300 ppm. Standard samples were prepared by diluting each 300 ppm solution with solvent (acetonitrile for thitsiol analysis and chloroform for urushiol analysis). In the same manner, solutions of lacquer samples from Myanmar and Korea were mixed at different weight ratios and vortexed for 5 min. Samples were injected into the HPLC instrument, using 20 µL for thitsiol analysis conditions and 10 µL for urushiol analysis conditions. For thitsiol analysis, acetonitrile and 20 vol.% aqueous acetic acid were mixed at a ratio of 9:1 and analyzed for 40 min using the isocratic elution method and 0.5 mL/min as the flow rate [13]. For urushiol analysis, acetonitrile and 0.1 vol.% aqueous trifluoroacetic acid were mixed at a ratio of 9:1 and analyzed for 20 min using the isocratic elution method and 1 mL/min as the flow rate [25]. Detection was performed using a UV detector at 260 nm (for urushiol analysis) and 280 nm (for thitsiol analysis) to obtain chromatograms.

## 4. Conclusions

In this study, HPLC was employed to quantitatively analyze the main component, thitsiol, particularly 3-(10-phenyldecyl) benzene-1,3-diol (thitsiol 16), in traditional lacquer from Myanmar obtained from *Gluta usitata*. Using thitsiol 16 and urushiol 15:2 standards as markers, two different mixed lacquer samples with different main components were analyzed after mixing Korean lacquer collected from *Toxicodendron vernicifluum* with Myanmarese lacquer, and the reliability of the quantitative analysis was investigated using calibration curves. Calibration curves were plotted by analyzing two different markers under different analysis conditions, and both showed high linearity. Furthermore, a blind test was conducted to explore the differences and particular characteristics of a lacquer based on its production area. Samples of mixed Myanmarese and Japanese lacquer and mixed Myanmarese lacquer and cashew nut shell liquid (CNSL) were analyzed using HPLC. This direct comparison allowed for the identification of any variations and provided insight into the distinct qualities of lacquer sap originating from different regions. Little interference between marker compounds was observed.

The outcomes of this study have practical implications for the restoration and preservation of lacquer-containing artifacts. Accurate identification of the origin of a lacquer within the lacquer industry can contribute to conservation efforts and provide valuable information for artisans and professionals working in the craft industry. Quantitative analysis of anacardic acid and cardol, a main catechol in CNSL, which is usually used with lacquer sap, will be studied for methodological application to real artifacts. Additionally, the identification of chemical structures and the mechanism of polymerization processes using ToF-SIMS will be carried out in future studies.

## Figures and Tables

**Table 1:** The retention time, response factor, limit of detection (LOD), and limit of quantification (LOQ) of the standards.

Compound	Retention Time [RT, min (±SD)]	Response Factor [R_f_ (±SD)]	LOD (ppm) ^1^	LOQ (ppm) ^2^
Thitsiol 16	20.44 (±0.31)	20.14 (±0.55)	0.24	0.79
Urushiol 15:2	11.40 (±0.28) 12.14 (±0.43)	17.48 (±0.33)	0.38	1.26

^1^ LOD = 3.3 × (standard deviation of 5 ppm standard/slope of calibration curve). ^2^ LOQ = 10 × (standard deviation of 5 ppm standard/slope of calibration curve).

**Table 2:** Contents of thitsiol 16 and urushiol 15:2 in lacquer samples.

Sample	Thitsiol 16 Content [wt.% (±SD)]	Urushiol 15:2 Content [wt.% (±SD)]
Myanmarese	7.00 (±0.260)	0.260 (±0.032)
Korean	0.208 (±0.050)	1.955 (±0.078)

**Table 3:** The mixing ratios of the blended lacquer samples that used for the calibration curves.

Blend	Myanmarese (wt.%)	Japanese (wt.%)	Blend	Myanmarese (wt.%)	CNSL (wt.%)
MJ01	0	100	MC01	0	100
MJ02	25	75	MC02	25	75
MJ03	50	50	MC03	50	50
MJ04	75	25	MC04	75	25
MJ05	100	0	MC05	100	0

**Table 4:** Quantitative analysis results of blind-tested samples.

	Myanmarese Composition [wt.% (±SD)]				
Blend	Myanmarese (wt.%)	Actual Content (wt.%)	Blend	Myanmarese (wt.%)	Actual Content (wt.%)
UMJ01	80.26 (±0.93)	80.0	UMC01	79.62 (±0.55)	80.0
UMJ02	61.35 (±0.47)	60.0	UMC02	59.75 (±0.11)	60.0
UMJ03	39.83 (±0.60)	40.0	UMC03	39.76 (±0.60)	40.0
UMJ04	20.84 (±0.79)	20.0	UMC04	19.79 (±0.37)	20.0

**Table 5:** The chemical formulae and structures of the standard compounds [28,31].

IUPAC Name (Trivial Name)	Structural Formula
3-(10-Phenyldecyl) benzene-1,2-diol (Thitsiol 16) C_22_H_30_O_2_ MW: 326.47	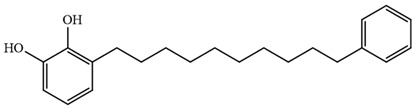
3-(8Z,11Z-Pentadecadienyl)-1,2-benzenediol (Urushiol 15:2) C_21_H_32_O_2_ MW: 316.48	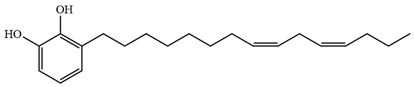

**Table 6:** Instrument conditions for HPLC measurements.

	Thitsiol	Urushiol
Column	C18 reversed-phase column (YMC-Pack Pro C18, 250 × 4.6 nm I.D. S-5 µm, 12 nm)	C18 reversed-phase column (YMC-Pack Pro C18, 250 × 4.6 nm I.D. S-5 µm, 12 nm)
Mobile phase	A: acetonitrile B: aqueous acetic acid, 20 vol% A:B = 9:1	A: acetonitrile B: aqueous trifluoroacetic acid, 0.1 vol% A:B = 9:1
Flow rate	0.5 mL/min	1 mL/min
Injection volume	20 µL	10 µL
Column temperature	30 °C	30 °C
Detection	UV detector, 280 nm	UV detector, 260 nm
Solvent	Acetonitrile	Chloroform
Analysis time	40 min	20 min
Elution mode	Isocratic elution	Isocratic elution

## Data Availability

Data are contained within the article.
